# The Enduring Effects of Medical Humanities on Medical Students: Short- and Long-Term Impacts of 15 Years of Teaching a Medical Humanities Course in a Swedish Medical Degree Program

**DOI:** 10.1007/s10912-025-09975-0

**Published:** 2025-08-30

**Authors:** Katarina Bernhardsson, Christopher Mathieu, Alexander Tejera, Lars Hagander

**Affiliations:** 1 Birgit Rausing Centre for Medical Humanities, Lund University, Lund, Sweden; 2 Centre for Languages and Literature, Lund University, Lund, Sweden; 3 Department of Sociology, Lund University, Lund, Sweden; 4 Department of Paediatrics, Department of Clinical Sciences in Lund, Skåne University Hospital, Lund University, Lund, Sweden; 5 Department of Paediatric Surgery, Department of Clinical Sciences in Lund, Skåne University Hospital, Lund University, Lund, Sweden

**Keywords:** Medical humanities, Medical education, Long-term follow-up, Professional development, Medical students, Medical doctors

## Abstract

This article analyzes the immediate and long-term effects of medical humanities teaching at a Swedish medical degree program. The objectives, format, and core pedagogical ideas and practices of an elective course in medical humanities are presented, situating the learning experience in the wider context of medical humanities in the Nordics. We conducted a qualitative, thematic analysis of course evaluations amassed over 15 years and of open-ended responses in an alumni survey sent out in 2023. Using these two sources, we compare the students’ immediate perception of medical humanities’ contribution to their education with what they discern when looking back. The students report that medical humanities teaching advances an understanding and responsiveness to narratives and furthers an ability to balance the rational, bio-medical perspective with a more holistic empathetic view of patients and illness, providing a deeper and broader toolkit to work from in clinical practice. The students perceive that they have acquired a specific expertise, obtained training in perspective taking, and yielded personal growth and agency. The interpretative sensitivities and competencies reverberated in the alumni survey and were reported to influence subsequent clinical work. Our study suggests that the impact of medical humanities teaching is transferred to both occupational practice and personal life, and that the impact is long term.

## Introduction

While interest in medical humanities is steadily increasing and its integration into medical and health profession education is gaining momentum, there is an ongoing debate about what medical humanities teaching can provide in terms of learning outcomes, and how best to implement the relevant teaching practices (Chiavaroli 2017; Coronado-Vázquez et al. 2023; Dalia et al. 2020; Ofri 2017). Two recent literature reviews have proposed frameworks for categorizing the epistemic functions of medical humanities teaching in medical programs (Dennhardt et al. 2016; Moniz et al. 2021a), but studies of medical humanities courses and perspectives outside Anglophone contexts are scarce.

This article analyzes short- and long-term impacts of medical humanities teaching on students at the Faculty of Medicine at Lund University, Sweden. At Lund University, medical humanities has become increasingly integrated into the medical degree program, with a curriculum of mandatory learning activities as well as in-depth elective options. This development started with the establishment of a 7.5-ECTS elective medical humanities course in 2008. In this article, the impact of the elective medical humanities course is analyzed through the students’ own words deriving from two data sources: course evaluations systematically collected over 15 years and an alumni survey conducted in 2023. Using these two sources, we compare students’ immediate perceptions of medical humanities’ contribution to their education with what they discern when looking back as practicing physicians. We also gauge the course’s perceived enduring impact on the doctors, their career trajectories, and their occupational practice.

## Background: The creation of an elective course in the medical program

Teaching medical humanities in medical education has a long history, yet the field has developed more recently in continental Europe. In the Nordic countries, medical humanities has primarily emerged since the 2010s (Bernhardsson and Hansson 2016), building on earlier traditions of health research in the humanities. Several research groups exist within medical humanities, and academic fields such as ethnology and history of medicine have taken an active interest in how medical humanities are congruent with their disciplines (Björkman and Söderfeldt 2023; Hansson and Irwin 2022). In Sweden, the early 2020s also saw the inauguration of three university centers with the designation “medical humanities,” so far the only ones in the Nordic countries.^1^

In the Nordic countries, the development of medical humanities in education is less prominent than in research, especially when it comes to learning activities embedded in professional health science programs. However, there is growing interest in educational aspects of medical humanities, giving rise to several initiatives, such as an elective course at Aarhus University (Vestergaard et al. 2013) and a mandatory narrative medicine course offered since 2017 at the University of Southern Denmark (Rasmussen et al. 2022).

The elective course analyzed here has been taught continuously since 2008, making it the longest-running medical humanities course in the Nordic countries. Earlier initiatives that preceded this course were extracurricular, so by bringing medical humanities into the curriculum of the medical program, the course broke new ground.^2^ The elective was established in close collaboration with Faculty of Medicine leadership and with strong student union support. Subsequently, medical humanities was integrated into the policy statement for the medical degree program in 2017.^3^ The start of the course coincided with revised national regulations for medical degree programs and with the revision of the program in Lund. From its inception, the elective course was regarded both as an initiative in its own right and as potential inspiration for further integration of mandatory medical humanities learning activities in the medical degree program and other health science programs.

The medical humanities course has offered an opportunity to bring in new perspectives, giving medical students access to underexplored aspects of their future profession and contributing more comprehensively to personal and professional development in what has been termed a “broader conception of professionalism” (Cooke et al. 2010, 60). An important point was to position the course not as purely individual *Bildung* but as *medical education*, i.e., positioning the humanities perspectives as intrinsic and necessary parts of a complete professional education.

The elective course offers perspectives from several arts and humanities disciplines, with literature and the arts being cornerstones. The timing of the course, placed toward the end of the last semester, means that the students address these perspectives in-depth just as they are about to enter their future profession, at a stage when they have considerable clinical experience to draw on and motivation to appreciate a broader range of perspectives.

The course’s elective status has consequences for both its design and our study. The students self-select, with approximately 10% of the 100–120 program students per semester choosing to enroll. This means that the students have an interest in medical humanities, with many exhibiting curiosity about the subject rather than previous expertise. Since the elective course is part of the program and not extracurricular, it has the same status, budget, and position in the curriculum as do other electives, such as global health, leadership, rheumatic diseases, plastic surgery, and healthcare economics.

The Swedish medical degree program is a five-and-a-half-year undergraduate program not requiring any prior university-level studies.^4^ Thus, most students come directly or almost directly from high school, without any designated pre-medical studies. In international comparison, the Swedish medical curriculum greatly emphasizes practical training, with three years of full-time clinical placements (Lindgren et al. 2011). Over the last decade, a national overhaul of medical education in Sweden has emphasized strengthening the program’s academic component through the Bologna Process, a stronger focus on progression, and an emphasis on active student participation. It also identified shortcomings in cross-disciplinary training and broader perspectives on medicine (Lindgren 2011). From the student perspective, this means that the medical humanities course offered within the program is many students’ first and only contact with university-level humanities. From the program perspective, this means that medical humanities has become part of the mission to bring a broader range of perspectives and academic disciplines into the curriculum.

### The core pedagogical ideas of the elective course

From its inception, the elective course has focused on the arts and on how central medical and caring themes are reflected and treated in art and literature. As Piemonte and Kumagai (2019, 44) highlighted, the arts can “offer answers to ‘experience-near’ questions,” and facilitate a kind of indirect teaching, presenting knowledge in other ways than students are used to, “thereby destabilizing students and opening them to new ways of thinking and seeing.” The arts thus become a catalyst for knowledge of experiences, contexts, emotions, and ways of seeing and thinking, paired with knowledge of their construction and representation in narratives and art. The arts also lay the foundation for discussions in the course group, in which students try out, analyze, and appraise different perspectives in relation both to works of art and to one another. Interpretative, hermeneutic dimensions are central to the course (see Meretoja 2017), and the educational interest in narrative is “interdisciplinary in both nature and application,” as Jones et al. (2014b, 5) emphasized. An important inspiration is Rita Charon’s work in narrative medicine (Charon 2006; Charon et al. 2017). The seminars also include the method of *shared reading*, i.e., joint reading of literature on site (Longden et al. 2015), and reflection through creative writing and the sharing of the resulting texts.^5^

The overall course aim has been to explore humanities perspectives to engage the students’ understanding and critical thinking regarding their profession, patient experiences, the doctor–patient relationship, the healthcare system, and the cultural, societal, and historical contexts of medicine. Building on the arts, the course includes perspectives such as the history of medicine, ethnology, medical anthropology, and linguistics. The three-hour class meetings include lectures by teachers from different disciplines followed by in-depth interactive seminars, often centered on a literary text or other art form. Themes cover, for example, the physician’s role today and historically, perspectives on death and dying, medicine as culture, personal narratives of illness, the physician as patient, historical perspectives on psychiatric diagnoses, the interrogation of emotions through film, conversation analysis of a doctor–patient encounter, as well as visual arts, medicine, and existential health.

The course is taught full time over five weeks, during which the students attend 14–16 class meetings and work on individual examination projects. Having courses concentrated in time, instead of running parallel with other courses over longer periods, is typical of Swedish university education. This concentration means that students devote themselves exclusively to the course, fully immersing themselves in it and in new ways of reading and discussing. The class meets almost daily, discussing the course reading material.

Although the course has developed over the years, with some lecturers and themes remaining constant and others varying over time, the core pedagogical ideas of the course design have remained consistent. To capture how these are expressed in the course, we highlight four aspects: the teachers and their expertise, the format of the seminars and the study environment, the syllabus, and finally the course examination.

First, the course has a number of affiliated teachers who have contributed to its thematic continuity over the years, including experts from the humanities and practicing physicians who combine their clinical experience with experience working in the humanities (Table 1). This mix of academic and medical expertise, half from medicine and half from the humanities, has been important from the start. The course convener’s fundamental expertise has been in literary studies, and the interactions between the teachers and the convener, who is present throughout the course, model interdisciplinary engagement. The instructors’ pedagogical and dialogical expertise and ability to impart knowledge and enthusiasm to the students have been crucial and as important as their subject expertise.

**Table 1:** The elective course in medical humanities

Title	Medicin som humaniora (Medicine as a humanities subject)
Credits	7.5 ECTS
Time	Five weeks full-time
Level	Advanced (Masters) level
Placement	The last five weeks of the 5.5-year medical degree program
Group size	Six to 12 students
Schedule	14–16 three-hour class meetings over five weeks
Course convener	Professor or associate professor in literary studies, who also supervises the examination projects
Teachers	Lecturers from the humanities and medicine. The lecturers from the humanities bring expertise in their subjects (i.e., literature, visual arts, history, ethnology, linguistics, anthropology, and creative writing) and specific interest in issues with medical relevance. The lecturers from medicine come from varying clinical specialties (e.g., primary care, oncology, and psychiatry) and have training in the humanities, some at the PhD level; some also have a background in the arts
Interdisciplinarity	Interdisciplinarity is a core feature of the course, manifested through the course themes, through the students meeting lecturers from different disciplines, and through the interaction between invited lecturers and the course convener, who is present throughout the course and co-leads the seminars
Teaching concepts	Narrativization: appreciating the human voice (e.g., of patients, patients’ relatives, and medical colleagues) in medical discussions through literature, autobiography, and film Historicization: studying historical documents to understand how medical perceptions have changed over time, for example, through reading psychiatric patient records from the eighteenth century Aestheticization: creating aesthetic experiences through artistic exposure and performance, through reading and studying art in the classroom and at cultural institutions Artistic creation: creating, sharing, and responding to one’s own and one another’s artistic creations
Examination	Oral examination of student projects in conference format, one to two days of 20-min presentations by the students, plus open discussion

Second, the formats of the class meetings and the study environment constitute key factors in creating an open and permissive discussion forum. The course convener plays an active part in creating space at interactive seminars, where the students bring their experiences and emotions to discussions and where different interpretations can meet. One way of creating this space is to blend the teacher’s and facilitator’s roles to encourage free discussion that can take directions the teacher was not expecting. The course schedule is freer than the students are used to, with more time for preparation, enabling different and more reflective kinds of reading than their usual fact-driven studies. This difference in study technique is also enhanced by excursions to museums and other venues.

Third, the syllabus contains academic texts from different disciplines as well as literary texts, film, and art. In the literary texts, the proximity to medical themes ranges from autobiographical illness experiences, via works in which medical themes are present or can be inferred, to works dominated by more general themes. The crucial feature is the lifelike and literary complexity of the chosen works, compelling the students to find multiple interpretations or more than one meaningful perspective to consider. As texts are chosen to bring out different voices and significant aspects in relation to the theme of the seminar, the length of the texts is also important, since the course is designed to encourage reflective reading and not to inadvertently compel fast and shallow reading. This means that full, complex novels are spaced out in the schedule, alternating with poetry, short stories, visual works, and films. When Virginia Woolf’s *Mrs Dalloway* has been part of the syllabus, it has been placed at the end of the course, providing the students a challenge and fostering insight into the competencies they have gained throughout the course. Other examples of course material are Agneta Klingspor’s autobiography about breast cancer *Stängt pga hälsosjäl* (Closed due to health reasons), short stories about his work as a rural doctor by Michail Bulgakov, poems by physician–poet Pia Dellson, Reidar Ekner’s long poem about his daughter’s death from cancer, *Efter flera tusen rad* (After several thousand rad^6^), portraits of doctors in Ingmar Bergman’s cinematic oeuvre, and the 2013 film *August: Osage County* depicting a highly complicated family dynamic that only partly concerns the illnesses portrayed.

Fourth, the examination is designed as an integrated and formative part of the course rather than being summative and evaluative. The examination does not require students to reproduce what has been taught, but encourages the creation of new knowledge and insights. The students are asked to conduct an in-depth study of a topic to be presented orally in a conference-like setting, with the course convener as moderator. The students choose the topic, the presentation mode, and whether they want to present their study in an artistic manner. This gives the students unusual freedom compared with conventional examination forms in the medical program. With the conference-like setting, the dialogic aspect of the course continues into the examination, and although the presentations are individual, the conference format creates a joint endeavor. The students learn from their peers as well as from their teachers, and the examination conference significantly expands the content of the course.

## Data and method

To bring out the students’ voices and the perceived enduring effects of the elective course, we conducted a qualitative thematic analysis of two different datasets.

The first dataset contains student responses in course evaluations written immediately after taking the course, collected systematically after all 24 course iterations over 15 years (2008–2022). These responses have a structured format with eight qualitative open-ended questions that the students could answer at as much length as desired. These questions have been phrased consistently over time (Appendix 1) and the responses have been collected by the course convener to improve the course and understand the interest in medical humanities among medical students. Course evaluations are common, often obligatory, in Swedish higher education but can take different forms. This evaluation was specifically designed to encourage nuanced and personal reflections, not asking for numerical ratings but rather narrative input. The free-text answers range from one to eight sentences per response. Of the total of 286 students of the course over 15 years, 93% completed the course evaluations, doing so in Swedish and anonymously.

**Fig. 1:**
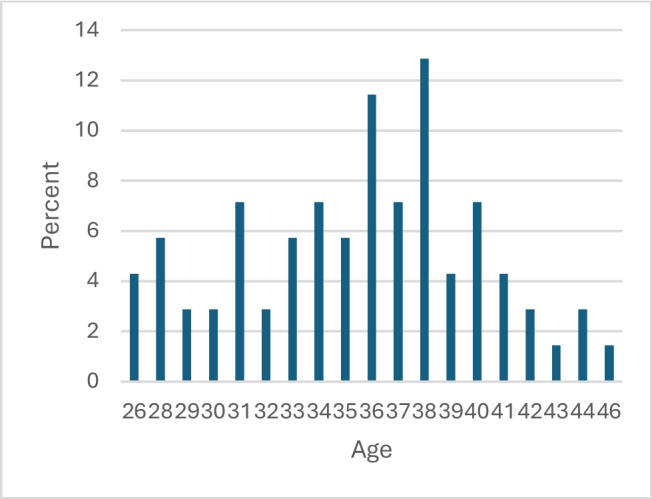
Respondents’ age at time of survey, years

**Fig. 2:**
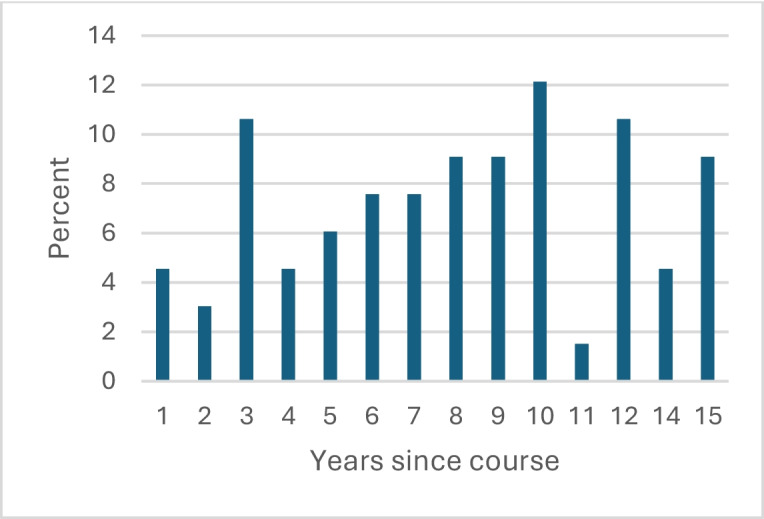
Years since the respondents took the course at time of survey

The second dataset is an alumni survey conducted in spring 2023 using both closed- and open-ended questions, the latter offering latitude for free-text answers of any length (Appendix 2) (the submitted free-text responses ranged from two sentences to a paragraph). It was conducted to allow the alumni to report on perceived enduring effects of the course on their medical practice and personal life. Links to the survey were sent by email to students for whom we could obtain valid email addresses (*n* = 213), by post to those for whom we could obtain only postal addresses (*n* = 26), and via LinkedIn (*n* = 22) to those we could only trace on that platform. Some individuals were approached via more than one contact method, for some we had more than one email address, and some individuals may have changed surnames or use aliases. We estimate that we contacted approximately 210 unique individuals (73%) of the 286 who had taken the course from 2008 to 2022. Three reminders were sent at three-week intervals. The response rate was approximately 33%, with 71 valid responses received. More than half (60%) of respondents were women, which is in alignment with the participants in the course (54% women). The average age at the time of completing the survey was 35.5 years, and respondents had participated in the course on average 8.1 years previously (Figs. 1 and 2). Responses were submitted by alumni from all years the course was offered. The survey interface allowed complete anonymity. The respondents were advised that participation was voluntary and that their responses would be used for both pedagogical development and academic research. All answers were collected and analyzed in Swedish and translated into English for publication purposes.

Through her work as a course convener, author 1 was, together with her predecessor, responsible for collecting the first dataset, and she has longitudinal insight into the pedagogical design, vision, and implementation of the course. As she has been associated with the course almost from its inception, a risk of bias from invested interest was identified. Therefore, the analysis of the first dataset was conducted by author 4 and of the second dataset by author 2, both of whom are not engaged in the course. All four authors have contributed to the study design, the alumni survey design, and the synthetic analysis for this article from the perspectives of their respective areas of expertise.

The qualitative method applied to both the course evaluations and the open-ended questions in the alumni survey conceptually followed Braun and Clarke’s (2006, 2019) phases of thematic analysis (see also King and Brooks 2017; Madill et al. 2010). This means that we first familiarized ourselves with the data, reading and re-reading the written texts, noting initial ideas, and highlighting interesting features, and then, through an active process, constructing, defining, and naming initial themes. For each question, similar answers were grouped together, providing an overview of the homogeneity and heterogeneity of responses. The groups of similar answers were then explored for tone, nuance, and complexity as well as frequency. Some respondents addressed only one topic in their open responses to a given question, while others addressed two or more topics to different degrees of depth or brevity.

Regarding the course evaluations, the themes were markedly consistent with the framework of Dennhardt et al. (2016), as further developed by Moniz et al. (2021a, 2021b). Dennhardt et al. (2016, 290) discussed the teaching of arts in medical education in terms of “art for mastering skills,” “art for interaction, perspective taking and relational aims,” and “art for personal growth and activism.” Although we initially familiarized ourselves with the data inductively without using predetermined themes, we have chosen to structure the presentation using the categories of the framework.

For the alumni survey results, we followed the pattern of the survey’s grouping of questions, first analyzing the enduring impact of the elective course in general, and then considering its impact on the alumni’s career trajectories and occupational practice. The alumni survey investigated the perceived personal and occupational impacts of the course and covered several topics (Appendix 2).

## The initial impact on the individual student

The initial impact of the course is studied through the qualitative analysis of student course evaluations completed immediately after the course iterations. In this analysis, three themes are discussed, structured according to the framework and categories presented by Dennhardt et al. (2016) in their scoping review, with minor modifications to reflect the students’ voices.

### Medical humanities help develop expertise

A common theme among the students was that the course helped them develop new expertise. The students reported that the teaching in medical humanities had provided something more profound than an enjoyable extra perk in their medical education. Some stated that the course had helped them rediscover and expand a humanistic core intrinsic to the medical field, while others considered medical humanities an epistemic addition to their medical curriculum. One indicative comment: “I wanted to activate another part of my brain—the reflective and interpretive part—that unfortunately so often has been constrained during our training, as we study like crazy.” This quotation points to a more personal kind of neglect and perceived blind spot that many students could identify in themselves and in their prior curriculum. Some students problematized these aspects even further, alluding to what others described as a hidden medical school curriculum and to the humanities course paradigm as a form of active recalibration (cf. Dennhardt et al. 2016, 71).

The course evaluations reflected the students’ curiosity and openness to expanding their medical scholarship to include insights from subjects such as the arts, history, anthropology/ethnology, and philosophy, and to considering how these subjects can be approached by physicians. The students considered the course a unique opportunity to broaden their academic education and to learn “how medicine can benefit from the humanities—and vice versa.”

The students expressed appreciation for their new knowledge, which they seemed to view as a kind of method or strategy to be deployed in the future: “A course that expanded our toolbox—now it is up to us to fill it.” Being positioned at the end of their medical training, the course was appreciated as a transitional hub, providing an opportunity for closure while giving new sets of skills as the students progressed to new beginnings as doctors.

### Medical humanities promote perspective-taking

A second theme conveyed by the students was the way teaching and learning in medical humanities promoted perspective-taking. Most of all, the students emphasized that the course provided new insights into illness and what it means to be ill: “Through literature we were able to augment our own experiences—we listened in on illness narratives and reflections and could see things from the perspective of patients.” They said that the course material and learning activities conveyed more holistic accounts of clinical encounters than did previous teaching, and that these helped challenge stereotypes and invoke a narrative sensitivity that complemented and refreshed their clinical awareness.

The students highlighted how the course discussions catalyzed not only patient-centered perspectives but also a better understanding of how their peers interpret clinical situations and what it means to be a doctor. It was “a course that strengthened our understanding and insight […] especially into others’ considerations of everyday situations we encounter as medical professionals”; it provided an opportunity “to observe the medical profession from an alternative viewpoint, to learn about our vocation through literature, art, and poetry.” The students thus viewed the humanities subjects as giving a sense of orientation and access to relevant perspectives on the medical profession: “Again and again, we concluded that medical professionals need other compasses to navigate, not just the biomedical one. The course has thoroughly restored my confidence in the possibility of remaining both humane and professional in my future career.”

### Medical humanities inspire personal growth and agency

A third dimension articulated by the students was how education in medical humanities inspired personal growth and agency. They described the course as a much-needed space for reflection and personal development that enabled them to reconnect with their professional core, “making me renew my understanding of why I want to become a physician.” For some, the course reinforced their choice of specialty, but an even more common experience was that the arts and literature helped them understand their aspirations: “I have gotten a clearer picture of who I want to become as a physician.” Another student underlined how the course provided a new entry point to becoming a doctor: “You probe your own emotions and empathy to be deployed in future encounters with patients and relatives. There has been nothing comparable so far in our education, and it can only be conveyed in this way.”

The students in the examined Swedish context did not emphasize medical humanities studies as inspiring societal activism (cf. Dennhardt et al. 2016; Moniz et al. 2021a). Why this was the case is something that could be explored further, considering whether this has to do with the course design, how healthcare inequalities are framed in Swedish society, or the structure of the Swedish universal, but decentralized, healthcare system. The students instead elaborated on how the course had inspired sensitivity to practical wisdom in a clinical setting, what Aristotle called *phronesis*. As one student wrote, “I now feel more prepared as a doctor to meet my patients and to meet myself, to deal with work’s complexity and its challenging and difficult aspects, to find strategies to relieve and cure patients, but also myself.” In this way, medical humanities perspectives provided a sounding board resonating with both the students’ theoretical knowledge and clinical experience: “The dual nature of clinical practice was a main reason I once decided to study medicine. To be allowed to focus on the humanistic side—this is something I have yearned for.”

The replies also illustrate how the students saw the course as fostering self-reflection, which could also take on an emancipatory aspect. One student related the course content to how they felt they had been formed by medical education:

The realization of how much I have changed, how much I have started to depreciate what is ‘irrational’ in life, how results oriented I have become. This new notion of human nature gradually sneaked up on me during medical training, it also included me, and maybe it was particularly strict with me, and resulted in this striving to become an objective instrument as a doctor.

This realization was then further associated with empathy, and more: “The humanities help me maintain empathy, not only for my patients but for myself. It serves as a reminder of wonder.”

Like Dennhardt et al. (2016), the students acknowledged a progression from perspective-taking to shifts in attitudes. Such a process aligns with the goals of the overarching university program description and is congruent with the national goal for Swedish medical programs, i.e., that students should demonstrate “self-awareness and the capacity for empathy, as well as a professional approach” (The Higher Education Ordinance 1993). However, there were also voices critical of too strong a focus on utility: “In a way, I find this quest for convincing arguments—why medics should engage in the humanities—somewhat trivial. Am I supposed to read literature to remain humane? Reducing the humanities to some confined set of expected learning outcomes—it is almost a paradox.” This student was protesting against a reductive focus on learning outcomes and seeing the value of medical humanities teaching as instrumental, instead emphasizing an intrinsic value unrelated to any specific utility. This comment is in line with a longstanding debate on literature in medical education, in which Anne Hudson Jones (1990) early on distinguished between an ethical approach, where literature functioned as a kind of “adjunct of medical ethics” without much attention to literary form, and an aesthetic approach, with a focus on literary and interpretative skills as clinically relevant knowledge. Even though the course primarily applies the aesthetic approach, the students’ comments show that the distinction between the ethical and aesthetic is not easy to make in practice, describing the course’s impact in words ranging from “empathy” and being “humane” to “understanding,” “wonder,” and insight into who they want to be as doctors.

## The lasting impact on the individual student: Perceived personal and occupational effects

### The alumni survey

The alumni survey investigated the perceived personal and occupational impacts of the course. In this section we focus on questions pertaining to three dimensions of such impact. The first concerns the impact of the course content and process on developing skills and perspectives bearing on the respondents’ working life and vocational practice, whereas the second concerns the same impacts, but instead bearing on the respondents’ non-occupational or private lives. The third dimension has to do with career trajectories. We also differentiate between normal times or everyday situations in working life and private life, on one hand, and exceptional circumstances, on the other, as the types of knowledge, understanding, and perspectives that come into play might differ depending on the circumstances (Swidler 1986).

### Enduring impact of the elective medical humanities course

Two open-ended questions probed the lasting impact of the course in general: “What have you taken with you from the course as a whole?” and “Is there anything in particular [from the course] that has left a lasting impression?” Answers to the first question brought out themes already seen in the course evaluations. These included developing a greater sense of empathy, a more nuanced understanding of the patients’ (and their relatives’) perspectives and experience, understandings of illness and health beyond the medical/biological, greater awareness of the psychological dimensions of medicine both beyond and formed by the lived experience of the body, and an increased capacity not just to think and feel but also to verbalize these topics. A typical response was: “Taking into consideration that the patient is part of a broader context than just the meeting at the clinic, s/he has an outlook and a truth that can help us understand the depth of a patient’s symptoms.” Another common theme in response to this question was being able to see and interpret contemporary medicine in its historical context and from a cultural perspective, and thereby being able to see its development (in positive and negative lights) and idiosyncrasies, as well as seeing the relationship and divergence between (medical) science and humanistic knowledges and forms of inquiry and expression. One respondent stated that the course gave “a perspective on modern medicine as an enormous deviation from how [medicine] has historically been.” A frequent comment referred to seeing and appreciating a more multifaceted physician role than that of a somatic mechanic, just diagnosing and treating the physical body. The following quotation sums up this ambition:

If we do not continue to have a soulful and intellectual connection to what we do in healthcare, then we easily run the risk as doctors of becoming the bureaucrats of the body, merely doing the bidding of the state and society, and not that of the individual human person.

This is more specific than simply the historical and cultural contextualization of medicine as it entails not just seeing a space for the use and expression of humanistic perspectives and skills in one’s own practice. Rather, it also highlights that this type of physician role is also experienced and practiced by others, creating an opening not just for seeing this type of practice but also for being able to explore this in dialogue with other physicians.

The second question elicited responses focusing on the format and execution of the course. Specific topics, texts, teachers, and activities were mentioned, but at a more general level the comments focused on three things. The first was the level of knowledge, competence, and engagement of the instructors, which was universally deemed exceptionally high. The second pertained to the examination, in which the students were able to freely choose a topic for an individual assignment presented to the class in a seminar format. In parallel to the comments on the high quality of the instructors and teaching, the students frequently commented on being impressed with the high quality of their fellow students’ projects, as well as the positive experience of being given a free and expressive opportunity to pursue a project addressing one’s own interest, and to share it. The latter dimension is part of the third and most commented on aspect of the course elicited by this question. What the students appreciated greatly about this course was that it provided a prompted, open, and stimulating forum in which to “discuss anything and everything, high to low” with one’s fellow students and instructors. This discussion opportunity was frequently said to be unique in their medical education and was characterized as open, vibrant, and vital.

A third question asked how the course could be improved. Aside from suggesting that the course could have been longer or offered earlier in the program, 6 of the 71 respondents offered substantive recommendations on improvements. Some students suggested that the range of artforms should be expanded, while others suggested the opposite: “With such a limited timeframe for the course, I think one should choose a limited number of perspectives and go deeper … rather than give an overview.” Other comments suggested explicit coupling or guidance on how course subjects can be integrated into work practice, especially regarding medical research, ethics, and work life sustainability.

### Impact on career trajectories

One question probed the impact of the course on career trajectories: “Did the course have an impact on your choice of specialization or other career choices?” About a quarter of the respondents (24%) reported that the course had influenced their choice of specialty. The majority thus replied that it did not, although some said that it reinforced a previously made choice, while others said that it helped push them toward specializations in which relational approaches were of higher value. Psychiatry and general practice were often mentioned in this context. One respondent, though, contested the idea that medical humanities lean more toward, or are most beneficial for, relational or more verbally oriented specializations:

There is, I believe, an image that these subjects [covered in medical humanities] are more important for primary care physicians (GPs) than, for example, anesthesiologists or surgeons. But this isn’t true. It shines through everywhere. When you as a surgeon must evaluate whether an abdomen should be operated on or not, you need to prepare the relatives to understand that we may be heading towards a situation in which the patient becomes brain dead, and that the patient could be a candidate for being an organ donor. Stitching a wound is easy. But behind each judgment question, where things must be weighed, is a deeper judgment where biology only contributes to half the evaluation.

Other respondents mentioned that the course made a decisive impact by encouraging them to engage in other activities, such as taking a break from medicine to go to art school before returning to medical school, engaging in a project creating children’s books about different medical conditions, studying other humanities courses in parallel to medical studies, or doing a PhD in medical history.

### Effects on occupational practice

A final set of questions focused on the perceived effects of the course on the former students’ occupational practice, on one hand, and private life, on the other, in both everyday and exceptional circumstances. Quantitatively, four out of five answered that they had benefited from the course content or perspectives in ordinary everyday practice at work (83%), and almost as many said that they had benefited in their personal life (76%). About a third reported that personal contacts made during the course had proved valuable in their occupational (28%) and personal (34%) lives.

Regarding everyday occupational practice, the responses circled back to the course, promoting greater insight into the situations and perspectives of patients and next-of-kin. For many the course also contributed to an ability to see and feel holistically. Another common theme was that the course helped them as physicians to deal with the sadness and despair that they can encounter in themselves and others, by giving them words, images, and references through which to approach and share these feelings. At the other end of the coping spectrum, some noted that the perspectives from the course gave joy, inspiration, and a greater passion for the job. A couple of the respondents spoke about how the course, in the abovementioned ways, changed their approach to patient encounters from being mere appointments to true *meetings*, in which the physician prepares to meet the whole person in front of her/him, seeing this as an important and special encounter between persons, and not just between the roles of physician and patient.

Regarding the difference between the contribution of the course in dealing with exceptional versus everyday occupational practice, a smaller proportion replied that they had benefited from the course content or perspectives in exceptional situations at work (35%) or in their personal life (17%). Many responded that their everyday work was already exceptional to a high degree and had difficulty differentiating between the two. Those who could differentiate reported situations of sudden and unforeseen fatality, and situations in which death simultaneously feels “entirely inexplicable and yet entirely natural.” Other exceptional circumstances were expressed in terms of feelings of inadequacy and loneliness. In these situations, the course afforded the ability to comfort and be comforted. It was also mentioned that the course assisted them in handling ethical dilemmas, and in seeing a wider and more historical picture.

Similarly to how some could not readily distinguish between everyday and exceptional circumstances at work, some respondents did not see a difference between themselves at work and in their private spheres. Other respondents viewed the course as contributing occupationally relevant skills and perspectives, and as also providing the foundations for a recuperative and recreational use and appreciation of the arts in their everyday lives. Some mentioned using the arts, and especially literature, as a means of escaping the burdens of work, and said that the course provided skills for appreciating the arts at a deeper level, providing more joy but also demanding more concentration, which created more relaxation and a greater buffer for work. The most common reflection was that the course led to a greater feeling of *Bildung* that pervaded their personal and occupational lives. As one person expressed it, the course led to “a richer inner life.” Regarding the contribution of the course to dealing with exceptional circumstances in one’s private life, responses again turned primarily on dealing with the illness, death, and loss of loved ones, but also on dealing with one’s own mortality. As one respondent wrote, “When I myself had a serious illness, it [i.e., the course] was invaluable for giving me the foundation to find comfort in the experience of others and also for me to be able to express myself.”

Finally, in response to a question about the future of medical humanities in their lives, almost all survey respondents considered continuous professional development in the subject to be occupationally valuable (96%). A large majority had already engaged in such activities (66%) and many expressed an interest in more (78%). In sum, practicing physicians exposed to medical humanities course saw enduring relevance in the approach and expressed a desire for opportunities to deepen their knowledge and exposure to it.

## Conclusion

Our analysis has explored the perceived effects of medical humanities on medical students in a Swedish context. The students reported in course evaluations that they had acquired specific expertise, obtained training in perspective-taking, and experienced personal growth and agency (cf. Dennhardt et al. 2016; Monitz et al. 2021a). This was corroborated by the alumni survey, in which we also saw how medical humanities learning could influence career trajectories and occupational practice. The analysis identified an impact on both the occupational and personal levels, supporting the combination of personal and professional development in medical education (Gordon 2003; Farahmand et al. 2022) and bolstering the claim that art and literature can play a part in this development (Charon et al. 2017; Jones et al. 2014a).

In the medical humanities literature, the debate on what is reasonable to demand of, or attribute to, medical humanities in educational settings has been lively. There is a tension between “the instrumental justification of humanities in medicine” and the intellectual practice of the humanities (Jones 2014, 27). Even though empirical studies show results, such as increased self-awareness and openness to other perspectives (Haidet et al. 2016), development of empathy (Graham et al. 2016), and better communication skills (Hammer et al. 2011), many proponents of medical humanities contest a simplistic view of the humanities as a way to become empathetic and humane, risking conflation of the terms “humanities” and “humanism.” While we agree with the caution against an oversimplified view of the effect of the humanities, our results also caution against the offhand rejection of a connection between the humanities and the latitude to reflect and gain new understandings, which the students in our study reported in both the short and long terms. In both the course evaluations and the alumni survey, a recurrent contrast was made between the cold, dispassionate, compartmentalizing, instrumental rationality that characterized the respondents’ medical education and the healthcare system they work in, and the warmth, passion, empathy, and holism that they found in the course, and that for many continued to inspire their *meetings* with patients. In the Swedish context of high-quality universal medical coverage, it is not surprising that the course evaluation and alumni survey respondents continuously took up the humane dimension, which is what they generally found lacking in the educational and healthcare systems. There was thus systemic criticism in the responses of the students, who found humanistic dimensions deficient in their training and practice. The course functioned to re-infuse these elements, reportedly with enduring effect.

The present results must be interpreted in the context of its study population and design. That the students responded uniformly positively to the open-ended questions of the study may reflect both overwhelming satisfaction and enthusiasm and/or self-selection in choosing the elective course and in choosing to complete the survey. The demographic distribution in terms of the age and gender composition of the study sample was congruent with the course’s student population, with a response rate of 93% and 33% for the course evaluation and alumni survey, respectively. In the course evaluations, some students indeed outlined areas for improvement, but these were course–managerial and technical in nature, typically related to the amount of compulsory reading required. The students also discussed at what time during the program compulsory medical humanities elements should be introduced. No agreement emerged: some argued that it should be integral from the outset of the studies, while others claimed that experience of clinical encounters was an essential prerequisite for appreciating medical humanities.

A strength of this study is that data collection took place over a period of 15 years, i.e., several generations of medical students, representing a varied and sequenced sample. The data encompass a large cohort of students, also surveyed in the alumni survey after entry into the profession to capture experience-based perceptions of long-term effects. From an inferential standpoint, it can be perceived as a limitation of this study that the course did not remain identical throughout the study period, as the course naturally developed over the years. However, the core pedagogical ideas of the course design remained consistent, and we arguably also found a consistency of impact despite slight variations in course content, which is validating and adds to the generalizability of our results.

Methodologically, the study is unique, as it is based on and compares contemporaneous assessments with long-term assessments of the course and its impact. The contemporaneous responses of students about to embark on medical careers are prospective and speculative regarding future utility, but based on concrete student experience. The alumni survey was completed by practicing physicians retrospectively reflecting on what enduring impact they had actually experienced from the course on their occupational and private lives.

The survey shows that the elective course created opportunities for expression and reflection perceived to be unique in the medical program in terms of topics, sophistication, and access to discussion partners, making a strong impression on most students. We believe that these effects reflect the course’s core pedagogical ideas, with the open form of the seminars and the examination concept affording room for explorative, reflective practice. The reading material in the syllabus prompted this exploration under the guidance of teachers with differing areas of expertise. It is worth noting that though we have summarized some typical responses here, many responses specifically dealt with singular and personal experiences. Though the course generally met with great appreciation, its impact naturally varied from person to person. Some dimensions were widely recognized and shared, while in other cases the course has had particular but profound effects on individual respondents, who may have found themselves in unique situations or have had the ability to use the course content and experience in unique and creative manners.

By comparing the retrospective survey with the course evaluations, we could see that the dimensions captured in the evaluations were still relevant from a long-term perspective. There was a self-reported enduring effect of participation in the elective course in occupational practice, especially of an interpersonal nature. In accordance with its goals, and as the students in the evaluations noted, the course equipped future doctors with a greater array of and more sophisticated tools with which to confront and communicate about core existential matters inherent in occupational and personal challenges. Many of the alumni have also found ways to further nourish and maintain their interest in medical humanities. Notably, and not insignificantly, the alumni also frequently mentioned the joy and perceptive sensibilities developed during the course.

This article contributes to the literature by exploring the content, process, and pedagogical concepts of a long-running elective course, and by analyzing students’ perceptions of medical humanities immediately after the course and after long-term retrospection. It draws on the pedagogical discussion of medical humanities and on sociological interest in its impact on occupational practice. Our results call for future theorizing and empirical research on how and why a relatively short course in medical humanities can influence students in the long term. Notably, we are currently working on an interview study with alumni of the course to address these questions. Importantly, the findings reported here indicate that several aspects of the immediate impact of medical humanities teaching on medical students stand the test of time, being sustained over years and in turn influencing skills, mindsets, and self-perceptions relevant to their professional development.

## Endnotes

^1^ The Swedish universities with established centers for medical humanities are Linköping, Lund, and Uppsala.

^2^ Medical humanities at Lund University can also be viewed from a longer-term perspective, dating back to the Vice Chancellor, surgeon, and art collector Philip Sandblom and his interest in art, creativity, and disease (Sandblom 1982).

^3^ The policy statement identified six core themes of the Medical Degree Program, which together support the student’s progression to becoming a “reflective doctor” (Björklund et al. 2020, 2) and positioned medical humanities as a subdivision under the “Professional Development” theme.

^4^ For new students since 2021, the medical program will be six years long, but still without requiring prior university-level studies.

^5^ For an in-depth discussion in Swedish of the teaching of literary seminars in this setting, see Bernhardsson et al. (2021).

^6^ “Rad” is here both the acronym for the unit “radiation absorbed dose,” which was used at the time, and the Swedish word for “line.”

## Data Availability

In accordance with research ethics regulations, the evaluations and survey material are not available to the public.

## Appendix 1: The questions in the course evaluation

Over the years, the course evaluation has consisted of eight questions, with only small revisions in the wordings. When the course was offered online due to the Covid-19 pandemic (spring 2020 to spring 2021), one extra question was added about the respondents’ experiences of the zoom instruction.

1. Medicin som humaniora is one of several elective courses you were asked to rank, taking into account your own interest. How did you rank Medicin som humaniora and what was your reasoning?
2. The course material has consisted of a course package with books, articles, and films. How do you assess the quantity and quality of the literature, and of the reading instructions for the seminars?
3. The course has been conducted with three forms of teaching, i.e., lectures, seminars, and supervision before the examination project, for 10–16 teaching hours a week. What are your views of this design, quantitatively and qualitatively?
4. Seminars and lectures have been distributed between the course convener and visiting lecturers from the humanities and medical faculties, respectively. Give your views of their quality, distribution, and coordination.
5. How would you generally characterize the course management, seminar environment, and social atmosphere during the course? Give some views of the positively/negatively influencing aspects.
6. For the examination, a special model has been tested, of collecting the examination projects in a conference. The participants have been faced with the task of making a conference presentation of approximately 20 minutes with subsequent questions and comments from the audience. The projects have been individual, but within a larger, common context. Give your views and personal experiences of the examination project.
7. The course is the only course in the program with a medical humanities focus. Do you think, with experience of the course, that such a focus is educationally relevant, i.e., justified and/or desirable, in the last semester of the program, or earlier in the medical program?
8. How would you like to describe your personal overall impression of the course?

## Appendix 2: The questions in the alumni survey

The survey started with demographic questions about what year the respondents took the course, what year they took their medical degree, gender, year of birth, current occupation and specialty, and the geographic area where they work. Subsequently, the questions were grouped under four headings. Yes and no questions had “yes/no” as options followed by a space for free-text answers, sometimes prompted by “How?,” “Why not?,” and “Please explain briefly.” All other questions had a space for free-text answers.

Impressions of the course.

1. What have you taken with you from the course as a whole?
2. Is there anything in particular that has left a lasting impression?
3. Is there anything you think was missing from the course?
4. Did the course have an impact on your choice of specialization or other career choices? If yes, in what way?

In professional life: use of the course’s content, perspectives, and networks.

1. Have you used the course’s content or perspectives in your everyday professional practice? How?/Why not?
2. Have you made use of the course’s content or perspectives in exceptional situations (rare, serious events) in your professional life? If yes, please explain briefly.
3. Did you get to know anyone in a more profound way during the course who has since then been important to you in your professional lfife?

Outside professional life: use of the course’s content, perspectives, and networks.

1. Have you used the course’s content or perspectives in your everyday life outside of your professional life? If yes, in what way?
2. Have you made use of the course’s content or perspectives in exceptional situations (rare, serious events) outside of professional life? If yes, please explain briefly.
3. Did you get to know anyone in a more profound way during the course who has since then been important to you outside of your professional life?

Continued interest in medical humanities.

1. Have you found ways to develop your interest in medical humanities after graduation, professionally or privately? How?/Why not?
2. Do you think that continuing education in medical humanities would be meaningful and fill a need in working life after graduation? How?/Why not? Do you have any ideas about how fully trained physicians could be given better opportunities to pursue an interest in medical humanities?
